# Tri‐reforming of Methane over a Hydroxyapatite‐Supported Nickel Catalyst Prepared by Cation Exchange

**DOI:** 10.1002/cplu.202500082

**Published:** 2025-06-30

**Authors:** Xuan‐Huynh Pham, Quoc‐Nghi Pham, Doan Pham Minh

**Affiliations:** ^1^ Centre RAPSODEE Université de Toulouse IMT Mines Albi, UMR CNRS 5302 Campus Jarlard Albi F‐81013 09 cedex France; ^2^ Université Paris‐Saclay CNRS, ICMMO 91405 Orsay France; ^3^ Sustainable Environment Research Institute Chulalongkorn University Bangkok 10330 Thailand

**Keywords:** cation exchanges, hydroxyapatites, nickel, trireforming of methane

## Abstract

Catalytic steam reforming of methane is a major process to produce synthetic gas (syngas), deployed at a large industrial scale. However, this is an energy‐intensive process since it is carried out at a high temperature above 900 °C with a large excess of water to preserve catalyst stability. Tri‐reforming of methane (TRM) is an alternative solution to steam reforming of methane, which allows valorizing not only methane and water but also carbon dioxide and oxygen, which are usually present in feedstocks such as biogas, landfill gas, or flue gas. Developing a highly performing catalyst for TRM under severe reaction conditions, in particular, with the inlet feeding composition close to the chemical stoichiometry, is meaningful to optimize process energy efficiency. In this work, a hydroxyapatite‐supported nickel catalyst containing 1 wt% Ni is synthesized by cation exchange and investigated in TRM. High activity and very good stability are obtained, making it among the most‐performing catalysts in TRM, in comparison with the data from the literature.

## Graphical abstract

A hydroxyapatite‐supported nickel catalyst containing 1 wt% nickel loading is successfully prepared by ion exchange, showing excellent catalytic performance in the trireforming of methane reaction at 800 °C and 1.4 bar.

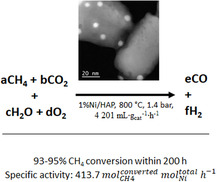

## 1 Introduction

The synthetic gas (syngas) is a gas mixture rich in CO and H_2_, which is an important platform gas mixture thanks to its various applications in chemical synthesis and energy fields.^[^ ^1^ ^]^ To date, syngas has mostly been produced from fossil resources, for example, coal, natural gas, and co‐products from petrochemistry.^[^ ^2^ ^]^ Using natural gas as feedstock, syngas has been produced at the large industrial scale via the catalytic steam reforming process, working at high temperatures (>900 °C).^[^ ^3^ ^]^ However, to preserve catalyst stability, a high molar ratio of steam to methane of around 3–4 is usually employed.^[^ ^4^ ^]^ Hot syngas should be cooled down before further downstream chemical synthesis processes, leading to the low energy efficiency of the global process.^[^ ^4^ ^]^ Tri‐reforming of methane (TRM) is a promising alternative solution, as recently discussed.^[^ ^5^ ^]^ In fact, in TRM reaction, methane can be reformed by a mixture of steam, carbon dioxide, and oxygen (oxidant agents) to produce syngas. This means TRM can be applied not only for the conversion of natural gas into syngas but also for the valorization of other feedstocks such as biogas and wastes such as flue gases from waste incinerators or fuel combustion units, as suggested by Song et al.^[^ ^6^, ^7^ ^]^ Moreover, TRM catalysts have usually been developed to reform a mixture containing a minimized excess of oxidant agents in comparison with the industrial steam reforming of methane.^[^ ^8^ ^]^ According to Pham et al.^[^ ^8^ ^]^ nickel is the most relevant active methane for TRM reaction, while a promoter such as platinum used at low loading (i.e., 0.5 wt%) has been recommended. About the supports, mixed oxides such as MgAl_2_O_4_ of CeO_2_–ZrO_2_ have been recommended.^[^ ^8^ ^]^ In addition, new catalyst supports such as hydroxyapatite (HAP, Ca_10_(PO_4_)_6_(OH)_2_) have also attracted attention. In fact, the first works of Alonso‐Perez et al.^[^ ^9^ ^]^ and Phan et al.^[^ ^10^ ^]^ on HAP‐supported nickel catalysts for TRM reaction revealed that these catalysts with conventional nickel loadings of 5–10 wt% were very active and stable in TRM at 800 °C. In these works, nickel has been deposited on the HAP surface by the classical incipient wetness impregnation or by the direct reduction of organometallic nickel precursor in ethanol solution, leading to the formation of nickel nanoparticles. In the present work, we develop HAP‐supported nickel catalyst with a low nickel loading of 1 wt% using cation exchange method, which is then investigated in the TRM reaction. HAP materials are well known by their ion exchange properties with several anions and cations, including Ni^2+^.^[^ ^11^ ^]^ Fixed on HAP support as isolated Ni^2+^ cations by this method, we expect that well‐divided nickel subnanoparticles and nanoparticles can be formed on the surface of the support after a thermal treatment step; these particles being favorable for methane reforming.

### 1.1 Results and Discussion

**Figure 1:**
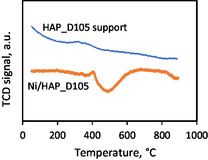
TPR profile of Ni/HAP_D105 and HAP_D105 support.

**Figure** **1** displays the temperature‐programmed reduction (TPR) profiles of the dried catalyst (Ni/HAP_D105) and of the dried support (HAP_D105). It is worth noticing that TPR analysis in this work recorded the thermal conductivity detector (TCD) signals, which took into account all the modifications of the outlet gas composition. The HAP_D105 support did not show reduction peaks, as expected for this nonreducible material. Some variations in the TCD signals of this material were probably due to the emissions of species such as CO_2_ and H_2_O during its heating, as previously reported in the literature.^[^ ^12^, ^13^ ^]^ In fact, during the thermal treatment of HAP, dehydration of hydroxyl groups and decarbonation of carbonate groups can take place, carbonate groups being incorporated into the apatitic structure of the hydroxyapatite support when it is exposed to the air.^[^ ^14^, ^15^ ^]^ For Ni/HAP_D105 catalyst, its TPR profile in Figure 1 was subtracted from the signal of the HAP support. Two reduction peaks could be observed. The first peak was small and centered at ≈380 °C, while the second peak was larger and centered at ≈510 °C. These peaks can be assigned to the reduction of nickel species that have strong interaction with the support,^[^ ^16^, ^17^ ^]^ or that are incorporated in the apatitic structure of the HAP support.^[^ ^16^, ^17^, ^18^ ^]^ However, the amount of reducible nickel species should be small, taking into account weak TCD signals of the reduction peaks. This could be explained by the fact that the nickel deposition by cation exchange led to the incorporation of nickel cations into the apatitic structure of the support according to Equation (1),^[^ ^14^ ^]^ which are difficult to be reduced under the operation conditions used.

(1) $${\text{Ca}}_{10}{\left({\text{PO}}_{4}\right)}_{6}{\left(\text{OH}\right)}_{2}{\text{+ xNi}}^{\text{2+}}\to {\text{ Ca}}_{10‐x}{\text{Ni}}_{x}{\left({\text{PO}}_{4}\right)}_{6}{\left(\text{OH}\right)}_{2}{\text{+ xCa}}^{\text{2+}}$$

**Figure 2:**
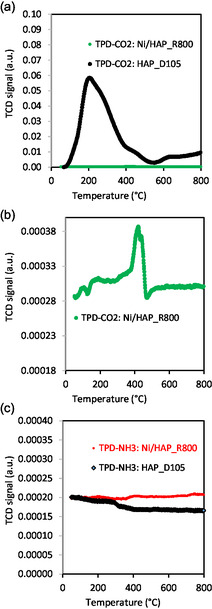
a,b) TPD‐CO_2_ profiles and c) TPD‐NH_3_ profiles of the catalyst reduced at 800 °C (Ni/HAP_R800) and of the initial support HAP_D105.

**Figure** **2** shows the temperature‐programmed desorption (TPD)‐CO_2_ and TPD‐NH_3_ profiles of the catalyst pretreated by reduction under H_2_ flow at 800 °C (Ni/HAP_R800) and of the dried support (HAP_D105). In Figure 2a, while the support HAP_D105 shows clear CO_2_ desorption peaks indicating the presence of basic sites, the catalyst reduced at 800 °C (Ni/HAP_R800) had any notable desorption peaks. This suggests a surface modification of the support during the nickel deposition by the cation exchange method. In fact, the pH of the initial nickel nitrate solution used for the cation exchange was around 3. During the cation exchange, the pH of the suspension progressively increased to around 6 (Figure S1 in Supporting Information), indicating a possible acid attack of the HAP support surface, which probably destroyed the basic sites of the support. In Figure 2b, the TCD signals of the Ni/HAP_R800 catalyst were zoomed, and we can see some traces of CO_2_ desorption at very low amounts. These peaks, found at relatively high temperatures (e.g. 420–440 °C), indicate the presence of basic sites with strong basicity strength.^[^ ^19^ ^]^

In Figure 2c, both the HAP_D105 support and the Ni/HAP_R800 catalyst did not show NH_3_ desorption peaks, suggesting a low density of acidic sites on the surface of these materials.

**Figure 3:**
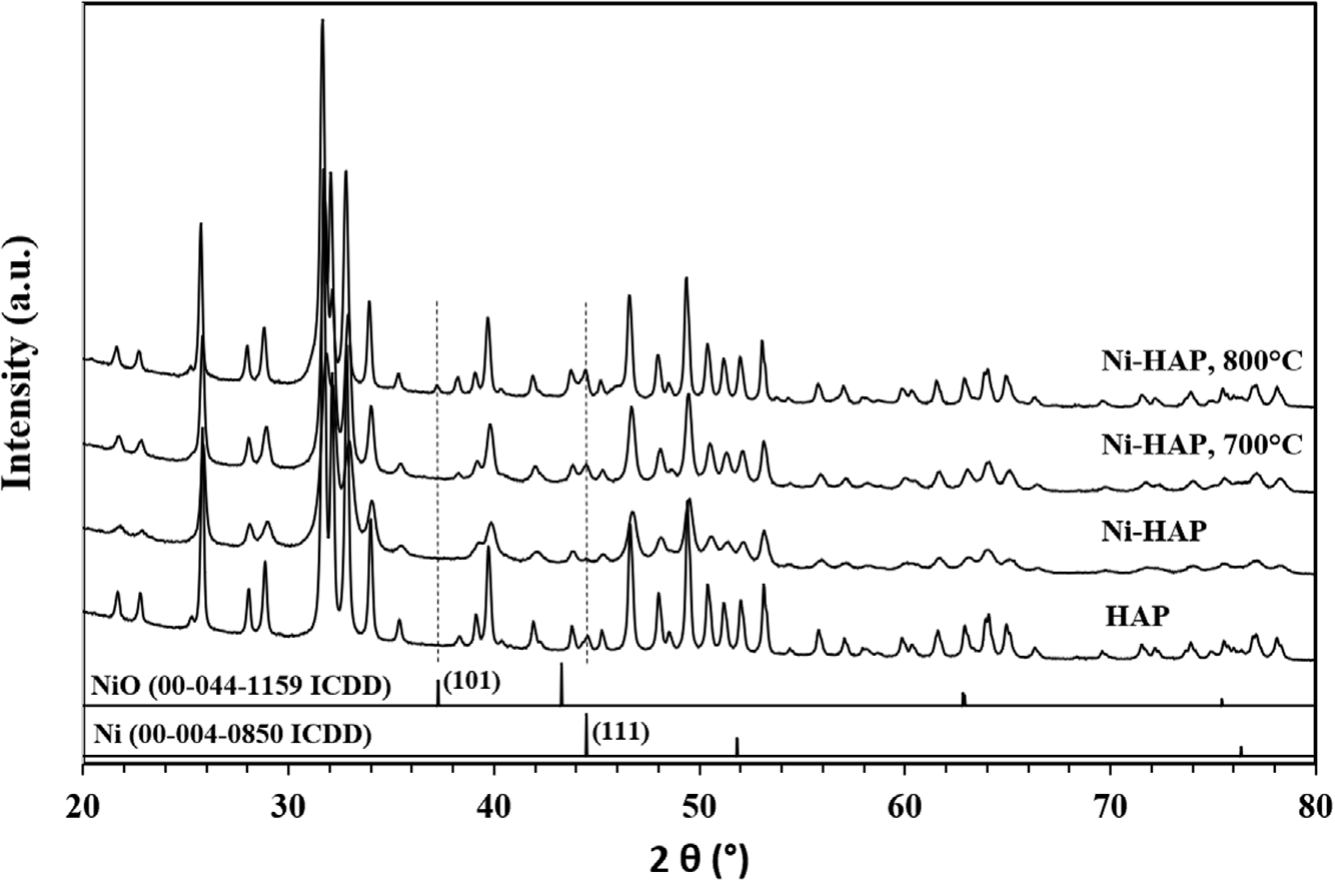
XRD patterns of fresh HAP supports, Ni/HAP, and Ni/HAP reduced under Ar/H_2_ (Ni/HAP_R700 and Ni/HAP_R800).

X–ray diffraction (XRD) patterns of fresh HAP, Ni‐HAP, and reduced Ni‐HAP (under 5vol.%H/x2082;/Ar at 700 or 800 °C for 2 h) are shown in **Figure** **3**. All samples display the typical reflections of crystalline hydroxyapatite (Joint Comitee on Powder Diffraction Standards (JCPDS) No. 09‐0432), confirming structural preservation upon Ni incorporation.^[^ ^12^ ^]^ Two main observations emerge: 1) peak broadening of HAP after Ni exchange, especially at 2*θ* ≈ 28.8° ([002] plane), indicating reduced crystallite size from ≈45 nm (fresh HAP) to ≈26 nm (Ni/HAP). This suggests indirectly the surface modification, likely due to acidic attack during ion exchange (see pH evolution of the suspension during ion exchange in Figure S1, Supporting Information), that is consistent with TPD results and literature values around 20 nm for HAP crystallites in Ni/HAP^[^ ^20^ ^]^; 2) no Ni or NiO peaks are visible in Ni/HAP, suggesting high dispersion or lattice incorporation of Ni^2+^.^[^ ^21^ ^]^ After reduction at 800 °C (Ni/HAP_R800 sample), a weak peak at 2*θ* ≈ 37.2° appears that can be attributed to NiO [101], with an estimated size of ≈38 nm. Finally, despite the low Ni content, a careful examination of the characteristic peak of Ni^0^ around 44.5° ([111] plane) allows confirming that Ni^0^ existed in the reduced samples, especially for Ni/HAP_R800 sample.

**Figure 4:**
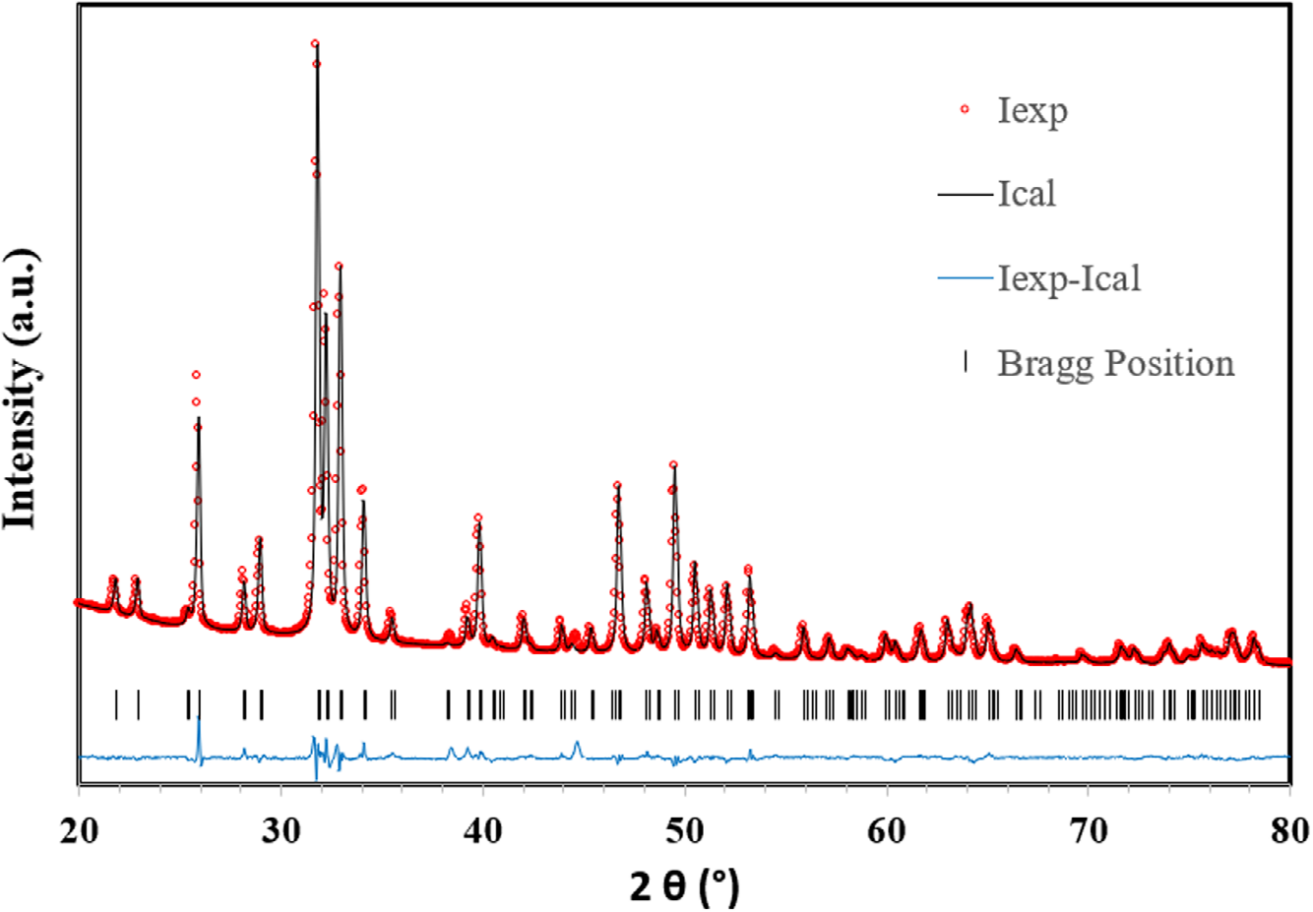
Rietveld refinement of XRD pattern for HAP support. The weighted‐profile and expected R‐factors are *R*_p_ = 8.69, *R*_wp_ = 9.16, *R*_exp_ = 2.26, *R*_Bragg_ = 3.18 (*χ* ^2^ = 16.40).

To further investigate the insertion of Ni^2+^ ions into the HAP lattice through ion exchange with Ca^2+^, Rietveld refinement of the XRD patterns was performed for both fresh HAP and Ni/HAP samples. As shown in **Figure** **4**, a very good fit was obtained using the hexagonal space group P6/x2083;/m (No. 176), with refined lattice parameters of *a* = *b* = 9.4218(3) Å and *c* = 6.8795(2) Å for HAP, in good agreement with literature values for stoichiometric hydroxyapatite.^[^ ^22^, ^23^ ^]^ For Ni/HAP, slight changes in lattice dimensions were observed (*a* = *b* = 9.414(1) Å, *c* = 6.8904(7) Å), while the unit cell volume remained nearly constant. Although Ni^2^+ has a smaller ionic radius than Ca^2^+ in the same coordination environment, low Ni content likely accounts for this minimal variation.

**Figure 5:**
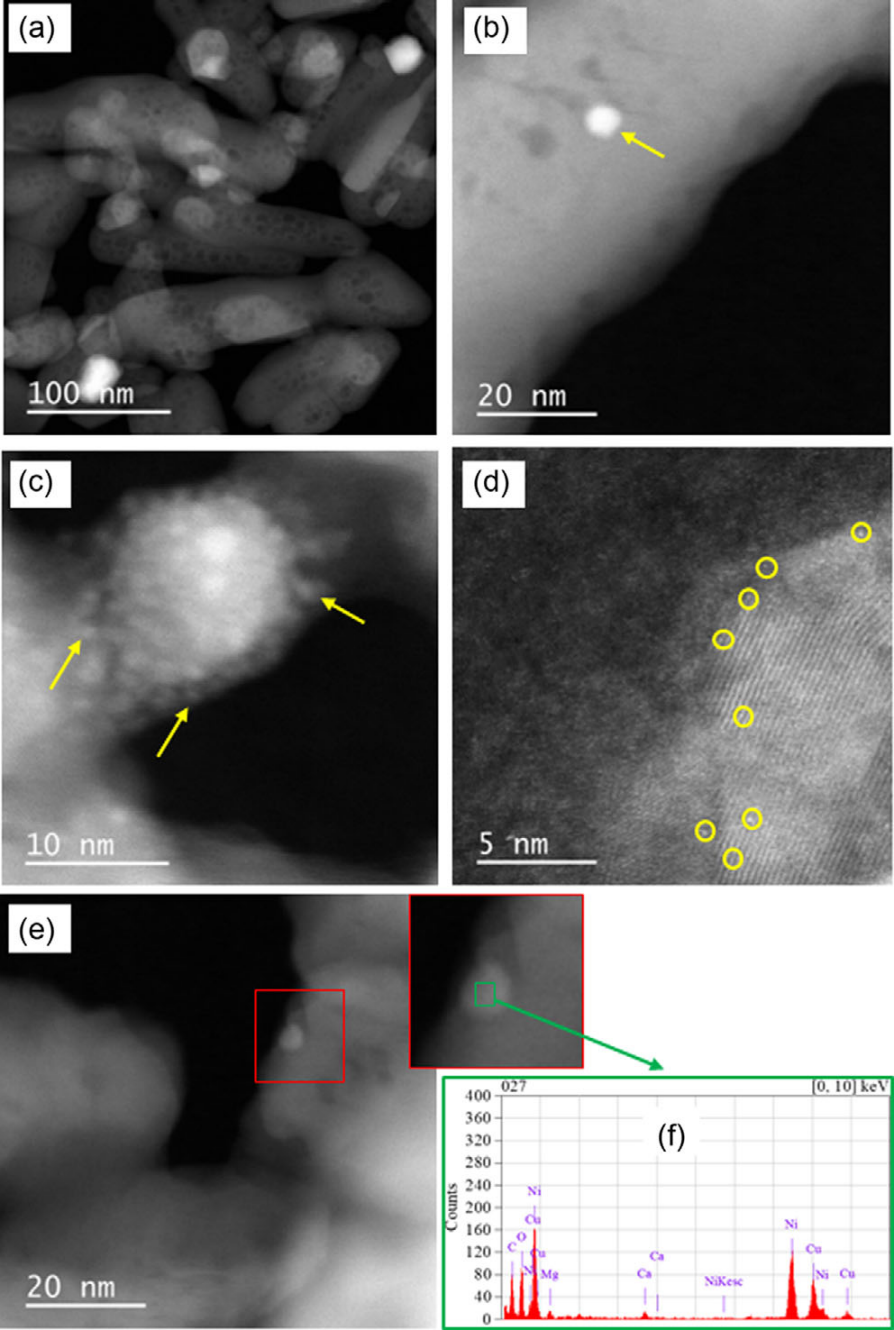
TEM images of the fresh Ni/HAP_R700 catalyst. Arrows and circles: examples of nickel particles.

**Figure 6:**
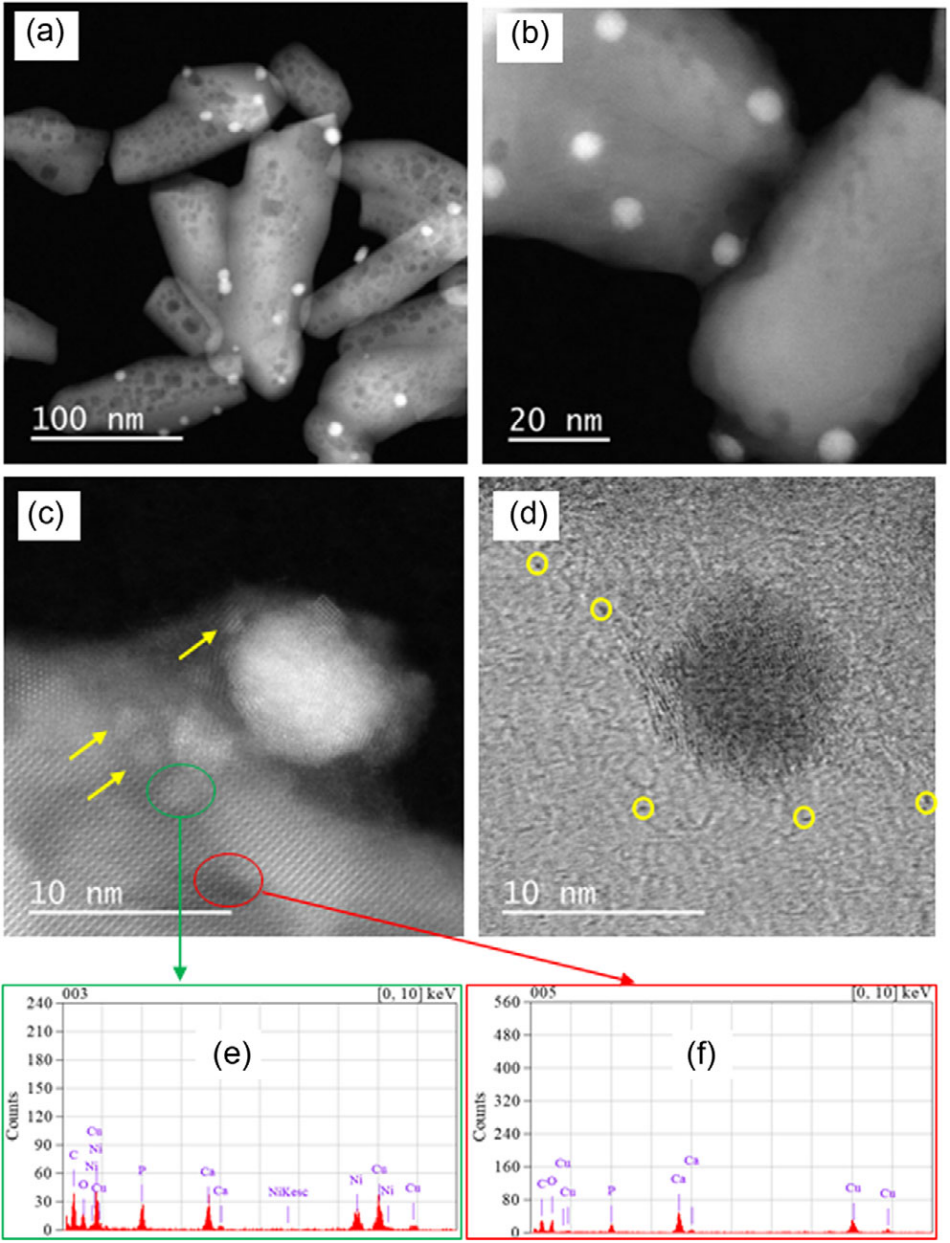
TEM images of the fresh Ni/HAP_R800 catalyst. Arrows and circles: examples of nickel particles.

**Figure** **5** shows transmission electron microscopy coupled with energy dispersive X‐ray analysis (TEM‐EDX) results obtained with the catalyst reduced at 700 °C (Ni/HAP_R700). Nickel particles of various sizes, from a few nm (Figure 5b, c) to dozens of nm (Figure 5a, Figure S2 in Supporting Information), were formed on the surface of HAP support, confirmed by EDX analysis (Figure 5e, f). Moreover, nickel subnanoparticles seemed also to be formed (Figure 5c). Similar results were also obtained with the catalyst pretreated by reduction under H_2_ flow at 800 °C (**Figure** **6**, Figure S3 in Supporting Information). Since nickel subnanoparticles exist, it is not evident to analyze the distribution of Ni particle size. Anyways, cation exchange is an efficient method for the deposition of Ni under the form of small nanoparticles and subnanoparticles on the surface of HAP.

**Figure 7:**
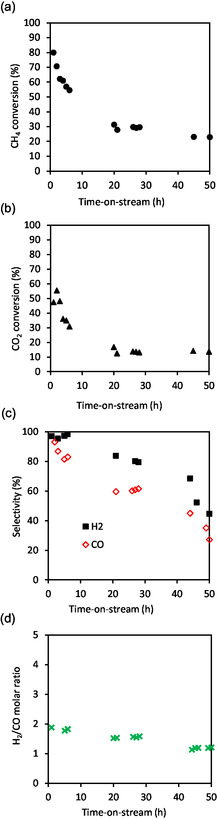
TRM reaction over Ni/HAP_R700. In situ reduction at 700 °C under 10%vol. H_2_/N_2_ for 2 h. Reaction conditions: 800 °C, total pressure: 1.5 bar, 340 mg catalyst, molar ratio of the feeding mixture: CH_4_/CO_2_/O_2_/H_2_O = 1/0.67/0.09/0.85, feeding flow rate of CH_4_: 45 mL min^−1^ (GHSV: 21006 mL g_cat_^−1^ h^−1^). a) Methane conversion, b) carbon dioxide conversion, c) carbon monoxide and hydrogen selectivity, and d) molar ratio of hydrogen to carbon monoxide.

**Figure 8:**
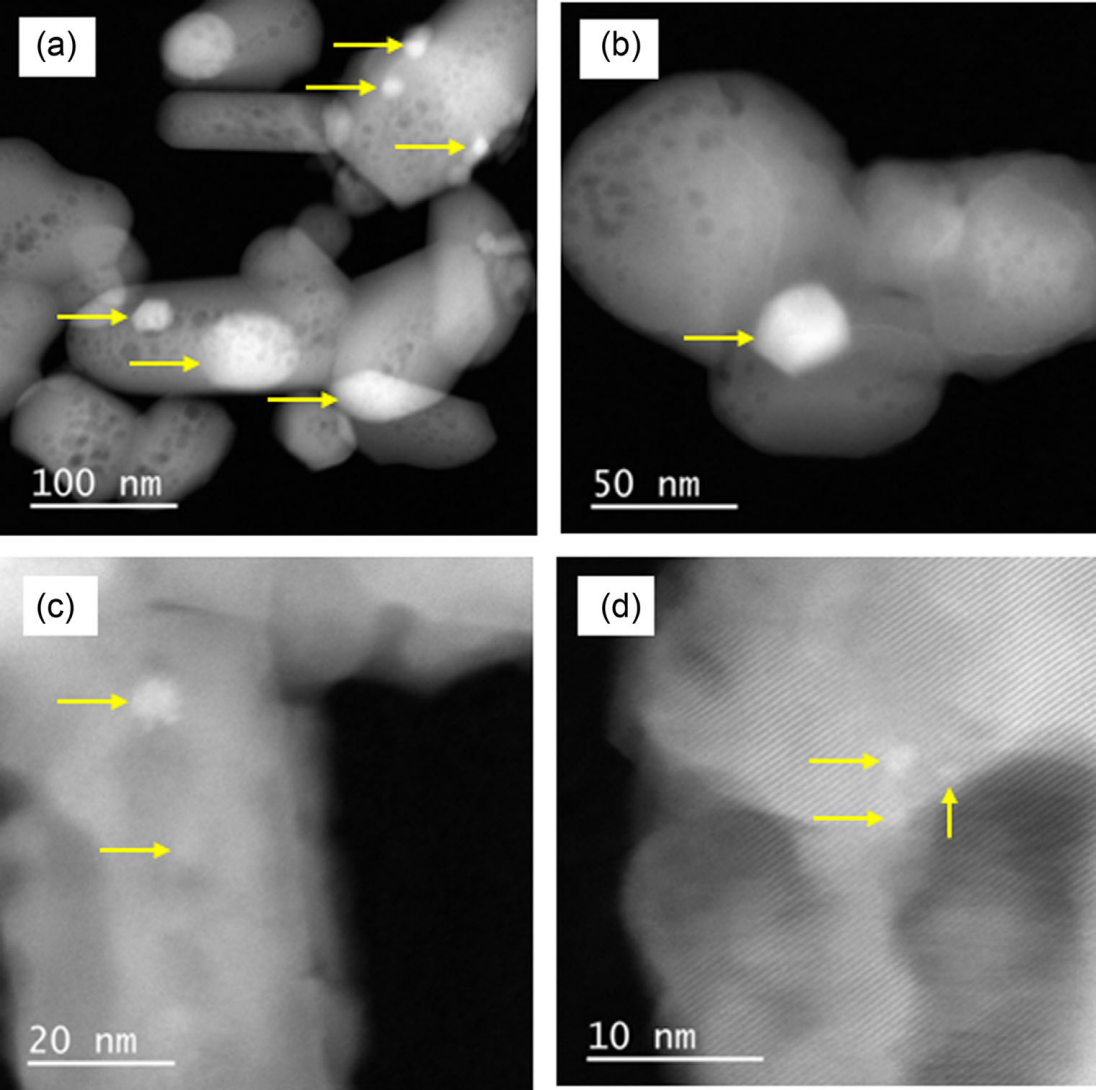
TEM images of the used Ni/HAP_R700 catalyst, recovered after 50 h‐on‐stream in TRM at 800 °C and 1.5 bar. Arrows: examples of nickel particles.

The catalytic activity of the catalyst reduced at 700 °C (Ni/HAP_R700) is presented in **Figure** **7**. The initial methane conversion reached 80%, which is very high for a nickel catalyst having a low nickel loading of 1 wt%. This initial methane conversion was only slightly lower than the one obtained with 5wt.%Ni/HAP prepared by incipient wetness impregnation under similar reaction conditions.^[^ ^10^ ^]^ However, this catalyst quickly deactivated within the first 24 h‐on‐stream. Then, the deactivation slowed down and seemed to be stabilized at around 20% after 50 h‐on‐stream. Similar trend was observed for carbon dioxide conversion, which decreased from around 60% to 10%. About the main products of the reaction, high selectivities in hydrogen and carbon monoxide were obtained at the beginning of the reaction. But these selectivities also decreased with the time‐on‐stream (Figure 7c). We could not explain these low selectivities into hydrogen and carbon monoxide for the moment, but it might be possible that other byproducts were formed and were not detected by the μ‐GC used. An important decrease in the molar ratio of H_2_/CO from 1.8 to 1.1 was also observed (Figure 7d). TEM analysis of the used catalyst recovered after the TRM reaction (**Figure** **8**) shows that thermal sintering seemed to take place. Large nickel nanoparticles up to around 30–40 nm appeared, in addition to small nickel nanoparticles, which were already observed in the fresh catalyst. This sintering could partially contribute to the deactivation of the catalyst. Subnanoparticles of nickel seemed to be no more present in the used catalyst.

**Figure 9:**
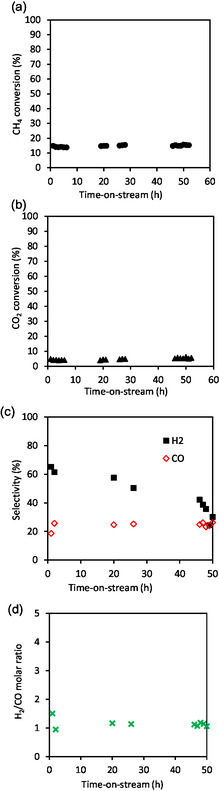
TRM reaction over Ni/HAP_R800. In situ reduction at 800 °C under 10%vol.H_2_/N_2_ for 2 h. Reaction conditions: 800 °C, total pressure: 1.5 bar, 340 mg catalyst, molar ratio of the feeding mixture: CH_4_/CO_2_/O_2_/H_2_O = 1/0.67/0.09/0.85, feeding flow rate of CH_4_: 45 mL min^−1^ (GHSV: 21006 mL g_cat_^−1^ h^−1^). a) methane conversion, b) carbon dioxide conversion, c) carbon monoxide and hydrogen selectivity, and d) molar ratio of hydrogen to carbon monoxide.

The catalytic behavior of the fresh catalyst reduced at 800 °C (Ni/HAP_R800) is presented in **Figure** **9**. The initial methane conversion was much lower than that observed in Figure 7 for the catalyst reduced at 700 °C. On the other hand, the catalyst is perfectly stable, even at a low methane conversion of around 15%. Similarly, carbon dioxide conversion was also stable at around 5% along the test. This result suggests that the catalyst could be well stabilized by a pretreatment at 800 °C under a hydrogen flux. Carbon monoxide selectivity was stable, but hydrogen selectivity decreased with time (Figure 9c), leading to a slight decrease in the molar ratio of H_2_/CO (Figure 9d). As previously observed in Figure 7 for the Ni/HAP_R800 catalyst, the selectivities into both hydrogen and carbon monoxide were relatively low. It is worth noticing that the catalytic behavior of Ni/HAP_R800 (reduced at 800 °C) is different from that of Ni/HAP_R700 (reduced at 700 °C). Taking into account the methane conversion profiles obtained with these two catalysts, it might be supposed that Ni/HAP_R700 still contains very active nickel species, which slowly disappeared during the catalytic test at 800 °C, causing the decrease in methane conversion to around 20% after 50 h‐on‐stream (Figure 7a). On the other hand, for Ni/HAP_R800 catalyst, the reduction under H_2_ at 800 °C might destroy these very active nickel species, and so the methane conversion was stable along the test.

**Figure 10:**
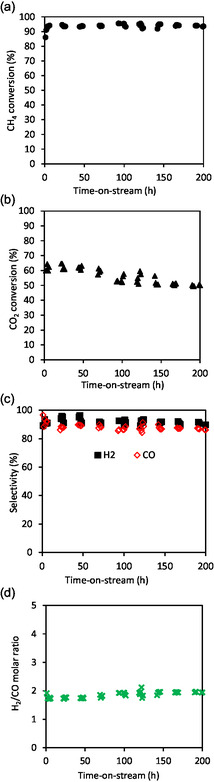
TRM reaction over Ni/HAP_R800. In situ reduction at 800 °C under 10%vol.H_2_/N_2_ for 2 h. Reaction conditions: 800 °C, total pressure: 1.5 bar, 1700 mg catalyst, molar ratio of the feeding mixture: CH_4_/CO_2_/O_2_/H_2_O = 1/0.67/0.09/0.85, feeding flow rate of CH_4_: 45 mL min^−1^. (GHSV: 4201 mL g_cat_^−1^ h^−1^). a) Methane conversion, b) carbon dioxide conversion, c) carbon monoxide and hydrogen selectivity, and d) molar ratio of hydrogen to carbon monoxide.

**Figure 11:**
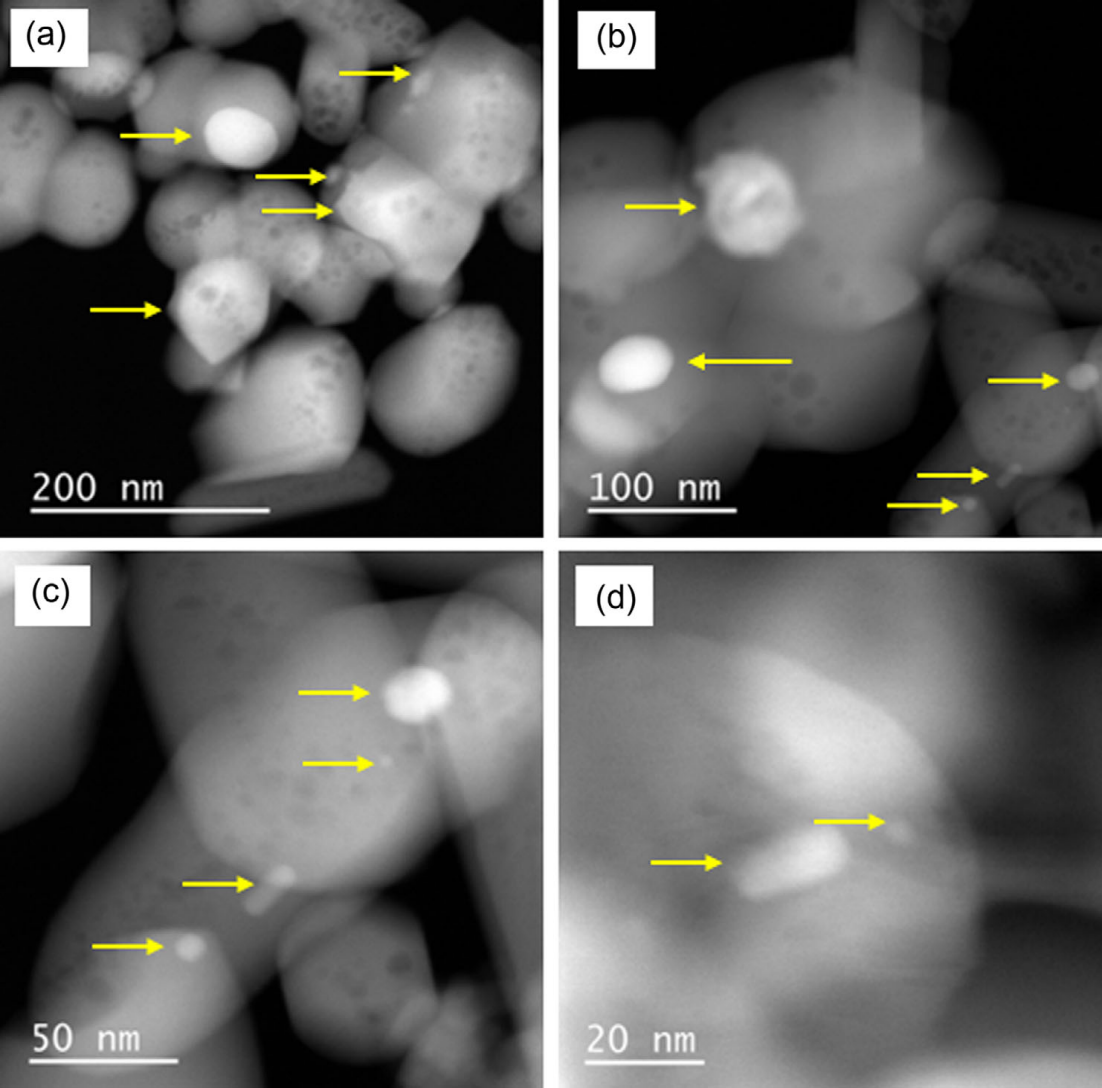
TEM images of the used Ni/HAP_R800 catalyst, recovered after 200 h‐on‐stream in TRM at 800 °C and 1.5 bar. Arrows: examples of nickel particles.

Taking into account the good catalytic stability of Ni/HAP_R800, another catalytic test was performed with this catalyst, with higher contact time by using a higher amount of catalyst. The results are presented in **Figure** **10** (see also the raw results of this experience in Figure S4 in Supporting Information). As expected, methane conversion reached around 93%–95% (which is slightly lower than the thermodynamic equilibrium, see Figure S5 in Supporting Information), and this conversion was kept stable at this value along the test of 200 h‐on‐stream. Carbon dioxide conversion slightly decreased from 60 to 50% (which is also slightly lower than the thermodynamic equilibrium, see Figure S5 in Supporting Information) after 200 h‐on‐stream. The selectivities in hydrogen and carbon monoxide were well stabilized at around 91%–95%, which led to a good stability of the molar ratio of H_2_/CO at around 1.8. In fact, the molar ratio of H_2_/CO only slightly increased, while the carbon dioxide conversion slightly decreased with the reaction time, which suggests that a reverse water–gas shift reaction also took place. TEM analysis in **Figure** **11** of the used catalyst recovered after 200 h of reaction at 800 °C confirms the phenomena observed in Figure 8. Notably, thermal sintering seemed to take place during the TRM reaction, leading to the formation of large nickel particles (Figure 11 a,b,c,d) in comparison with those of the fresh catalyst. However, the good catalytic stability in Figure 10 suggested that this thermal sintering should happen at the beginning of the reaction. The high catalytic performance of Ni/HAP_R800 catalyst could be attributed to the formation of small nickel nanoparticles, stabilized by strong interaction of exchanged nickel species with the HAP surface.

**Figure 12:**
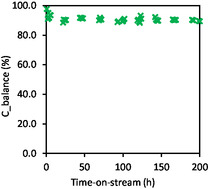
Carbon balance (ratio of total outlet carbon determined by μ‐GC to total inlet carbon) during TRM reaction over Ni/HAP_R800. In situ reduction at 800 °C under 10%vol.H_2_/N_2_ for 2 h. Reaction conditions: 800 °C, total pressure: 1.5 bar, 1700 mg catalyst, molar ratio of the feeding mixture: CH_4_/CO_2_/O_2_/H_2_O = 1/0.67/0.09/0.85, feeding flow rate of CH_4_: 45 mL min^−1^. (GHSV: 4201 mL g_cat_^−1^ h^−1^).

Temperature‐programmed oxidation (TPO) analysis was also done to verify the formation of solid carbon after the catalytic test. The used Ni/HAP_R800 catalyst recovered after 200 h‐on‐stream has been submitted to heating within 30–900 °C under 5 vol% O_2_/N_2_ (Figure S6 in Supporting Information). No significant change in the TCD signal could be recorded, suggesting that solid carbon was not significantly formed on the surface of the Ni/HAP_R800 catalyst in TRM reaction at 800 °C. This is in accordance with the previous results reported in the literature.^[^ ^10^ ^]^ The TPO result allows better understanding of the stability of this catalyst observed in Figure 10. Furthermore, from the μ‐GC analysis results, the carbon balance (see Equation (6)) could also be established and is presented in **Figure** **12**. During 200 h‐on‐stream, the carbon balance was found within 89%–98%, which was well buckled.

To gain insight into the surface chemistry relevant to the catalytic activity of the reduced Ni‐based materials, particularly concerning the transformation of Ni^2+^ incorporated in the HAP structure into Ni nanoparticles, X‐ray photoelectron spectroscopy (XPS) was employed. Because of the complexity in Ni 2p spectra, due to multiple splitting, shake‐up satellites, and charge effects, a particular deconvolution procedure was performed, using reference Shirley line shapes. These references were derived from high‐reliability measurements of standard compounds, metallic Ni, NiO, and Ni(OH)_2_, acquired on the same spectrometer under identical conditions. This approach ensured a robust interpretation of the surface chemical states of nickel as it transitions from the HAP structure to form metallic nanoparticles.

**Figure 13:**
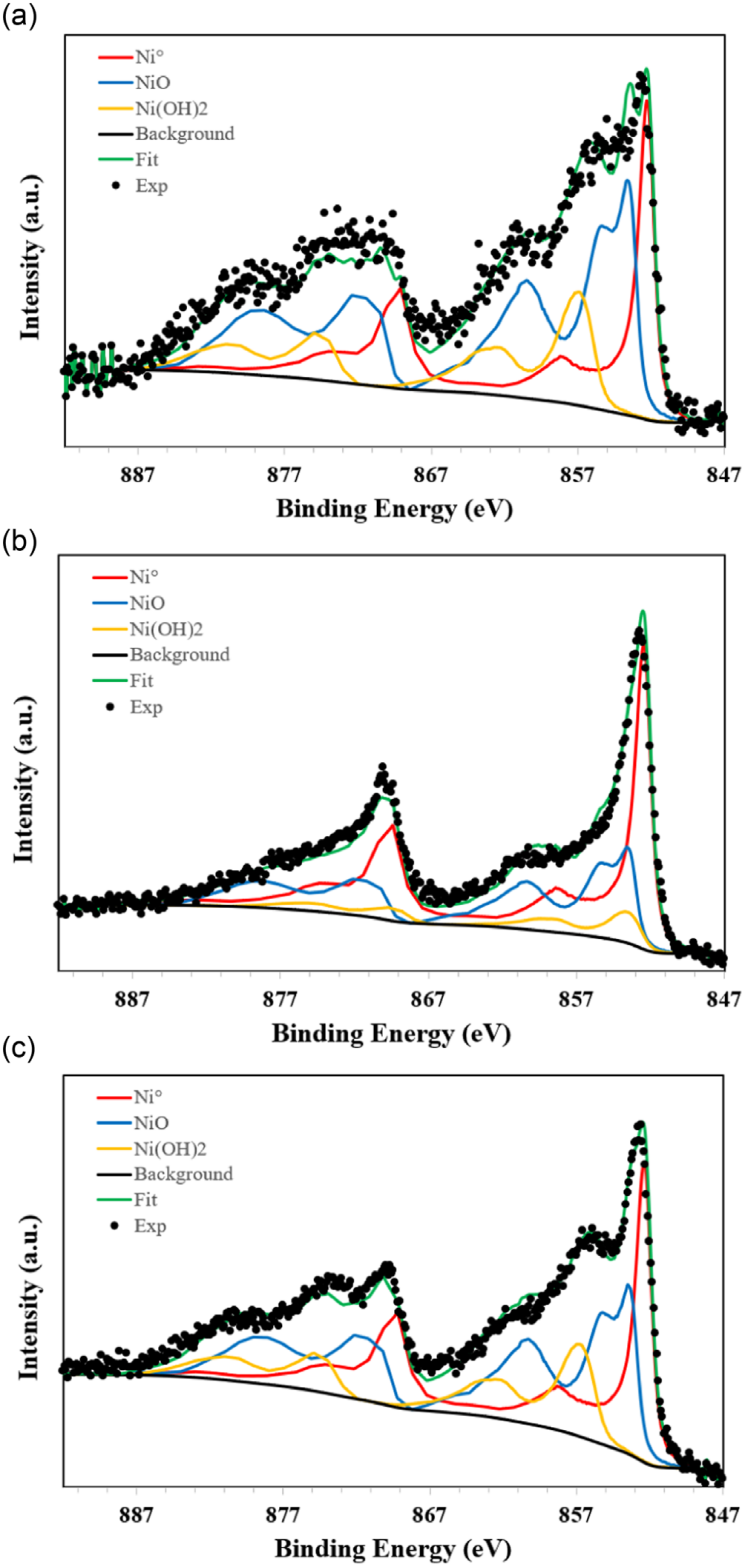
XPS deconvolution of the Ni 2p region for reduced catalysts: a) Ni/HAP_R700 point 1, b) Ni/HAP_R700 point 2, and c) Ni/HAP_R800.

For reduced Ni/HAP catalysts, the deconvolution of the Ni 2p_3/2_ region for both Ni/HAP_R700 and Ni/HAP_R800 clearly reveals three distinct contributions: metallic Niº (≈852.3 eV), NiO (≈853.6 eV), and Ni(OH)_2_ (≈856.9 eV), as shown in **Figure** **13**. The presence of metallic nickel in both samples indicates that the majority of Ni^2+^ initially incorporated into the HAP lattice was successfully reduced and migrated to the surface. The appearance of NiO and Ni(OH)_2_ is attributed to surface oxidation during sample handling, a phenomenon commonly observed in air‐sensitive metallic systems.

Interestingly, although all samples were derived from the same synthesis batch, XPS analyses performed at multiple surface points revealed spatial variations in Ni distribution. For Ni/HAP_R800 sample, the XPS window was consistent across the sample surface, indicating a relatively homogeneous Ni dispersion (34.3% metallic Ni° of the total Ni content). In contrast, the Ni/HAP_R700 sample exhibited marked heterogeneity: some areas showed predominantly oxidized nickel species (as an example: 29.3% metallic Ni° in Figure 13a), while others displayed a strong metallic Ni signal (as another example: 52.7% metallic Ni° in Figure 13b). This observation suggests that the reduction at 700 °C was less effective or less uniform than at 800 °C, potentially due to local variations in HAP crystallinity or Ni dispersion. This hypothesis could explain the difference in catalyst properties between Ni/HAP_R700 and Ni/HAP_R800, as mentioned above.

In addition, the surface Ca/P atomic ratio in these samples, as determined by XPS, ranged from 1.4 to 1.5, which is lower than the theoretical value of 1.6 for stoichiometric HAP.^[^ ^23^ ^]^ This suggests a calcium‐deficient surface, likely due to partial substitution by Ni^2^+ or leaching during the exchange step. This observation is consistent with the XRD results.

**Table** **1** compares the results obtained with those reported in the literature under similar reaction conditions of temperature and pressure. However, other conditions, including the inlet composition, the contact time, the catalyst mass employed, and the reactor size are not close to each other. Both methane and carbon dioxide conversions varied in large ranges, which should be linked to the physicochemical properties of each catalyst. From the inlet methane flow rate, methane conversion, catalyst mass, and nickel loading, the specific activity (SA, 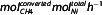) could be calculated. This calculation took into account the total amount of nickel and not the amount of surface nickel since this information was not systematically available. The comparison of SA values in Table 1 shows that the catalyst developed in this work is situated among the best‐performing TRM catalysts. More globally, HAP‐supported nickel catalysts prepared by cation exchange (Ni/HAP_R800, Table 1, this work) or by incipient wetness impregnation (5Ni/HAP,[10] Table 1) are among the most efficient catalytic systems for TRM reaction.

**Table 1:** Comparison of some results reported in the literature with those of this work under similar conditions.

Catalysts	Ni loading [wt%]	T [°C]; P [bar]; cataly]t mass used [mg]	Molar ratio of CH_4_/CO_2_/O_2_/H_2_O/N_2_	GHSV [mLg_cat_^−1^ h^−1^]	CH_4_ conv. [%]	CO_2_ conv. [%]	TOS [h]	SA [ 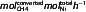 ]	
Ni/HAP_R800	1	800; 1.4; 1700	1/0.67/0.09/0.85/0	4 201	93	50–64	200	413.7	This work
5Ni/HAP	4.0	800; 1.4; 340	1.0/0.67/0.1/0.9/0	21 202	90	35–50	300	494.2	[10]
Ni@HAP	10.1	800; 1.4; 340	1.0/0.48/0.09/0.78/0	14 900	90	60	70	185.4	[9]
RNAl_M‐53_	13.6	800; 1; 1000	1/0.5/0.1/0.0025/1	3 135	98	37.5	100	22.7	[27]
Ni/Al_2_O_3_	10(a)	800; 1; 100	1/0.23/0.07/0.46/0.28	17 220	76	20	10	169.0	[28]
Ni/CeO_2_‐ZrO_2_	10(a)	800; 1; 100	1/0.23/0.07/0.46/0.28	17 220	49	14	10	109.9	[26]
Ni/MgO	10(a)	800; 1; 100	1/0.23/0.07/0.46/0.28	17 220	24	7	10	52.8	[26]
Ni/SBA‐15	10(a)	800; 1; 100	1/0.23/0.07/0.46/0.28	17 220	67	19	10	149.9	[26]
Ni/TiO_2_	10(a)	800; 1; 100	1/0.23/0.07/0.46/0.28	17 220	36	11	10	80.3	[26]
Ni/ZrO_2_	10(a)	800; 1; 100	1/0.23/0.07/0.46/0.28	17 220	63	16	10	139.5	[26]
NiCH	6.7	800; 1; 200	1/0.33/0.16/0.33/0	48 000	75	40	5	659.9	[29]
NiC0.4 H	8.1	800; 1; 200	1/0.33/0.16/0.33/0	48 000	60	38	5	436.7	[27]
NiC1H	7.3	800; 1; 200	1/0.33/0.16/0.33/0	48 000	50	25	5	403.8	[27]

a) Targeting nickel loading.

## 2 Conclusion

HAP‐supported nickel catalyst with low nickel loading of 1 wt% has been successfully synthesized by cation exchange method. Nickel nanoparticles and subnanoparticles of various sizes could be observed by TEM. TPD analysis mostly showed the presence of basic sites of the HAP support used. TPR analysis revealed the existence of several nickel species which were reducible at different temperatures under hydrogen flow. XRD and XPS highlighted the transformation of Ni2+ species, that are previously fixed on HAP surface, into Ni nanoparticles after H_2_ reduction. Furthermore, the catalyst pretreatment by reduction under hydrogen highlighted the importance of the selected reduction temperature. The catalyst pretreated at 700 °C had a high initial catalytic activity in TRM at 800 °C, but it quickly deactivated, while the one pretreated at 800 °C showed very good catalytic stability at both low and high methane conversion. This catalyst was found to be among the best‐performing catalysts for TRM reaction, in comparison with the data from the literature. Further work would focus on the improvement of nickel dispersion (e.g., obtention of principally nickel subnanoparticles) and on the stabilization of divided nickel subnanoparticles (e.g., by using sintered HAP support) to design an optimal TRM catalyst.

## 3 Experimental Section

3.1

3.1.1

##### Catalyst Preparation

A stoichiometric HAP was provided by an industrial partner. This material was produced from the precipitation of Ca(OH)_2_ and H_3_PO_4_ acid and had a specific surface area of 60 m^2^ g^−1^. Its molar Ca/P ratio reached 1.67 as revealed by elemental analysis by Inductively Coupled Plasma Atomic Emission Spectroscopy (ICP‐AES). Nickel nitrate hexahydrate, Ni(NO_3_)_2_6H_2_O, of analytical purity grade, purchased from Fisher Scientific, was used as nickel precursor.

Nickel deposition on HAP support was performed by cation exchange method. Thus, 20 g HAP were dispersed in 1 L of an aqueous solution of Ni(NO_3_)_2_6H_2_O (25 g L^−1^) under stirring (400 rpm) for 6 h, where part of Ca^2+^ on the surface of HAP was replaced by Ni^2+^.^[^ ^24^ ^]^ Then, the solid was filtered and washed 10 times with distilled water and finally dried overnight at 105 °C. This catalyst is called Ni/HAP_D105, which had a similar specific surface area than the virgin HAP support. The elemental analysis by ICP‐AES showed the Ni content reaching 1.01 ± 0.01 wt%. From this dried catalyst, two other materials were obtained after reduction under 5%H_2_/N_2_ at 700 and 800 °C for 2 h, which were named thereafter Ni/HAP_R700 and Ni/HAP_R800, respectively.

##### Catalyst Characterizations

TPR was performed with a Micromeritics Autochem 2920 equipped with a TCD. The dried catalysts were heated under a flux of 5%H_2_/N_2_ (10°Cmin^−1^ heating rate). TPD was also performed with this Micromeritics Autochem 2920 equipment. First, the dried catalyst was reduced at 800 °C under a flux of 5%H_2_/N_2_ (10 °C min^−1^ heating rate). Then, it was cooled down to 100 °C under He. At this temperature, the reduced catalyst was saturated with a flux (50 mL min^−1^) of 5%CO_2_/N_2_ (TPD‐CO_2_) or 5%NH_3_/N_2_ (TPD‐NH_3_). After purging, the desorption was carried out by heating the sample under a flux of He (50 mL min^−1^) up to 800 °C (10°Cmin^−1^ heating rate). TCD signals were monitored during the desorption step. TPO was done with used catalyst recovered after the catalytic test, using the same Micromeritics Autochem 2920 equipment. The sample was heated from 30 to 900 °C (5 °Cmin^−1^ heating rate) under 5 vol.%O_2_/N_2_ flux (50 mL min^−1^) to record any change in TCD signal by complete oxidation of solid carbon.

The XRD patterns were recorded on an X’PERT PRO MDP from Philips PANalytical in the 2*θ* range of 20–80 degrees with an increment of 0.039 degrees and an acquisition time of 240 s per step. A Cu tube was used as the X‐ray source, operating at an intensity of 40 mA and a tension of 45 kV, with a fast detector (X'celerator). The FullProf program^[^ ^25^ ^]^ was used to perform the Rietveld refinement method.^[^ ^26^ ^]^ The XPS spectra were recorded by a Thermo Fisher Scientific (Courtaboeuf, Les Ulis, France) spectrometer equipped with an Al Kα monochromatic high‐energy radiation source (*hv* = 1486.7 eV) and a hemispherical analyzer operating in Constant Analyzer Energy (CAE) mode. XPS data were analyzed using CASA XPS software version 2.3.25PR1.0 (Clearwater, FL, USA). Survey scans were conducted with a pass energy of 200 eV and a step size of 1 eV. High‐resolution windows were acquired with a pass energy of 50 eV and a step size of 0.1 eV. To minimize the charge effect, a higher current (480 μA) was applied. For XRD and XPS measurements of Ni/HAP samples reduced under Ar/H_2_ flow at a high temperature, specific precautions were taken to minimize the rapid oxidation of metallic Ni upon exposure to air.

TEM coupled with energy‐dispersive X‐ray analysis (TEM‐EDX) was performed with a JEM‐ARM200F Cold FEG Field Emission Gun microscope.

##### TRM Reaction

**Figure 14:**
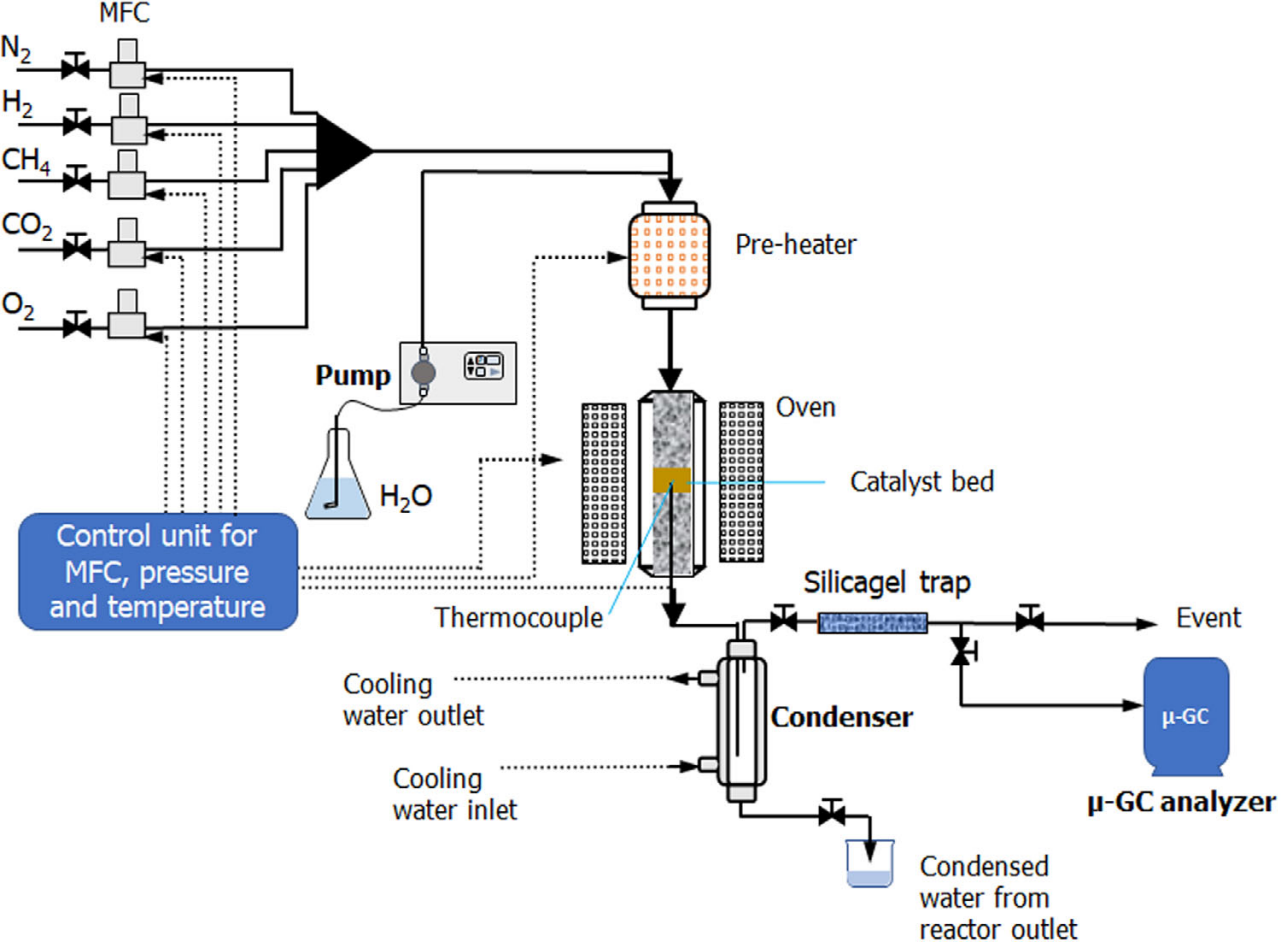
Scheme of the catalytic reactor for TRM reaction.

Catalytic tests in the TRM reaction were performed with a fixed‐bed reactor (8 mm inner diameter), which is heated by an electric oven. **Figure** **14** shows the scheme of the reactor. The flow rate of the feeding gases was controlled by mass flow controllers (MFC), while the flow rate of water was controlled by a precision pump. Liquid water was evaporated by the preheating oven at 120 °C, and this steam was mixed with CH_4_, CO_2_, and O_2_ to produce the feeding mixture with the desired composition. In this work, the molar ratio of the feeding mixture was fixed at CH_4_/CO_2_/O_2_/H_2_O = 1/0.67/0.09/0.85, with the flow rate of CH_4_ equal to 45 mL min^−1^. The feeding mixture passed through the catalyst bed where the catalytic reaction takes place. The catalyst bed was set at the center of the tubular reactor, while the rest of the tubular reactor was occupied by inert alumina powder, sintered at 1000 °C with a specific surface area of 2 m^2^ g^−1^. A thermocouple was set at the center of the catalyst bed to control the reaction temperature. Prior to the TRM reaction, the catalyst was in situ reduced under a flux of 10%vol. H_2_/N_2_ (70 mL min^−1^) at 700 °C (for Ni/HAP_R700 catalyst) or 800 °C (for Ni/HAP_R800 catalyst). At the outlet of the reactor, the residual steam was condensed by a condenser at 5 °C. Trace of steam in the gas from the outlet of the condenser was trapped in a silica gel tube at room temperature. Periodically, the total amount of water in the condenser and in the silica gel tube was weighed to determine the water outlet flow rate. The permanent gases (CH_4_, CO_2_, CO, O_2_, H_2_) at the outlet of the water trap were analyzed by a μ‐GC (MyGC model, Agilent). The total flow rate of the permanent gases was measured with a gas counter. From the results of μ‐GC analysis, the conversion and the selectivity of the reaction could be calculated according to Equation (2) to (5).

(2) $${\text{CH}}_{4}\;\text{conversion:}\;X_{{\text{CH}}_{4}}\left(\%\right)\frac{Q_{{\text{CH}}_{4}}^{\text{inlet}}-Q_{{\text{CH}}_{4}}^{\text{outlet}}}{Q_{{\text{CH}}_{4}}^{\text{inlet}}}\times 100$$

(3) $${\text{CO}}_{2}\;\text{conversion}\;X_{{\text{CO}}_{2}}\;(\%)=\frac{Q_{{\text{CO}}_{2}}^{\text{inlet}}-Q_{{\text{CO}}_{2}}^{\text{outlet}}}{Q_{{\text{CO}}_{2}}^{\text{inlet}}}\times 100$$

(4) $$\text{Selectivity}\;\text{into}\;H_{2}\text{:}\;S_{H_{2}}\;\left(\%\right)=\frac{Q_{H_{2}\;\text{formed}}}{2\left(Q_{{\text{CH}}_{4}}^{\text{inlet}}-Q_{{\text{CH}}_{4}}^{\text{oulet}}\right)+Q_{H_{2}O}^{\text{inlet}}-Q_{H_{2}O}^{\text{oulet}}}\times 100$$

(5) $$\text{Selectivity}\;\text{into}\;\text{CO:}\;S_{\text{CO}}\;\left(\%\right)=\frac{Q_{\text{CO }\;\text{formed}}}{Q_{{\text{CH}}_{4}}^{\text{inlet}}{-}_{{\text{CH}}_{4}}^{\text{oulet}}+\;Q_{{\text{CO}}_{2}}^{\text{inlet}}-Q_{{\text{CO}}_{2}}^{\text{oulet}}}\times 100$$

(6) $$\text{Carbon}\;\text{balance:}\;C_{\text{balance}}=\frac{TC_{\text{μ‐GC}}^{\text{outlet}}}{TC^{\text{inlet}}}\times 100$$

where $Q_{{\text{CH}}_{4}}^{\text{inlet}}$ and $Q_{{\text{CH}}_{4}}^{\text{outlet}}$: methane inlet and outlet flow rate (NmL min^−1^), respectively; $Q_{{\text{CO}}_{2}}^{\text{inlet}}$ and $Q_{{\text{CO}}_{2}}^{\text{outlet}}$ carbon dioxide inlet and outlet flow rate (NmL min^−1^), respectively; $Q_{H_{2}O}^{\text{inlet}}$ and $Q_{H_{2}O}^{\text{outlet}}$: water vapor inlet and outlet flow rate (NmL min^−1^), respectively; $Q_{\text{H2 formed}}$ and $Q_{\text{CO formed}}$: hydrogen and carbon monoxide outlet flow rate (NmL min^−1^), respectively; $TC_{\text{μ‐GC}}^{\text{outlet}}$: Total outlet carbon quantified by μ‐GC (mol min^−^^1^); and $TC^{\text{inlet}}$: Total inlet carbon (mol min^−^^1^).

## Conflict of Interest

The authors declare no conflict of interest.

## Supporting information

Supplementary Material

## Data Availability

The data that support the findings of this study are available from the corresponding author upon reasonable request.
