# Supplementary material for: Tri‐reforming of Methane over a Hydroxyapatite‐Supported Nickel Catalyst Prepared by Cation Exchange
Source: Chempluschem. 2025 Jun 30;90(8):e202500082. doi: 10.1002/cplu.202500082 (PMC12352719; doi:10.1002/cplu.202500082)
Supplement: Supplementary file 1 — Supplementary Material [file CPLU-90-e202500082-s001.pdf]

Supporting Information

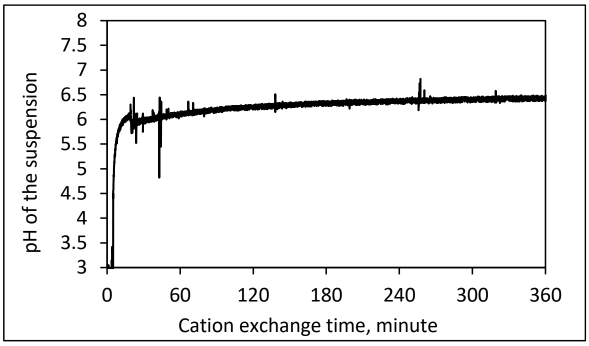

Figure S 1. pH evolution during the nickel deposition by cation exchange method.

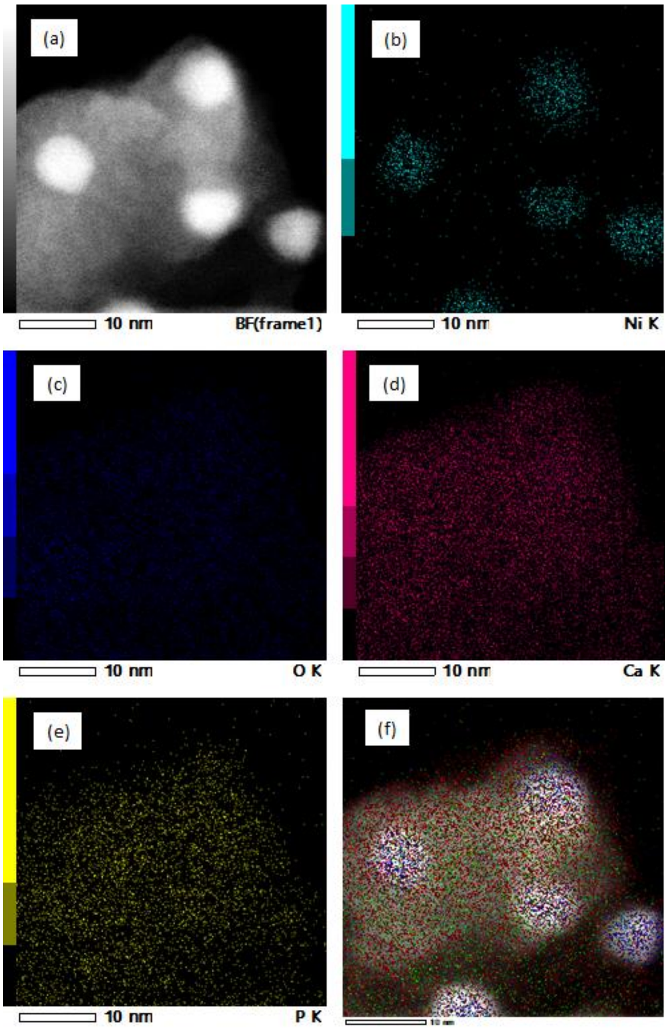

Figure S 2. Element mapping by TEM-EDX of the fresh Ni/HAP_R800 catalyst: (a) mapped zone; (b), (c), (d), (e): distribution of nickel, oxygen, calcium, phosphorus, respectively; (f): element overview.

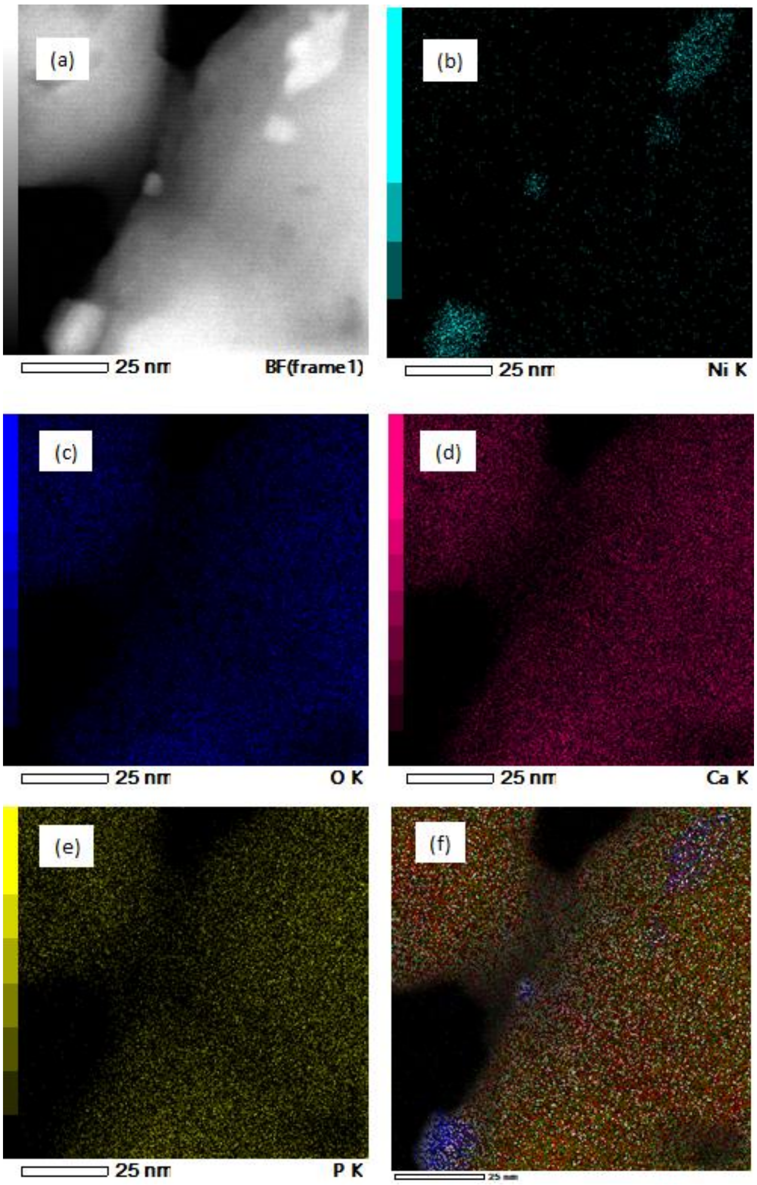

Figure S 3. Element mapping by TEM-EDX of the used Ni/HAP_R800 catalyst, recovered after TRM test at 800 °C for 200 hours-on-stream: (a) mapped zone; (b), (c), (d), (e): distribution of nickel, oxygen, calcium, phosphorus, respectively; (f): element overview.

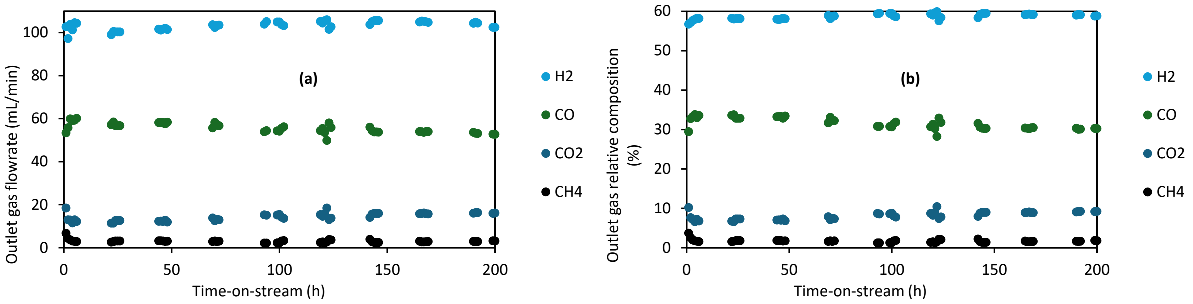

Figure S 4. (a) Outlet gas flowrate (mL/min) and (b) outlet relative gas composition (%) of the TRM reaction over Ni/HAP_R800. In-situ reduction at 800 °C under 10%vol.H_2/N_2 for 2 h. Reaction conditions: 800 °C, total pressure: 1.5 bar, 1700 mg catalyst, molar ratio of the feeding mixture: $CH_4/CO_2/O_2/H_2O$ = 1/0.67/0.09/0.85, feeding flowrate of CH_4: 45 mL·min^{-1}. (GHSV: 4201 mL·g_{cat}^{-1}·h^{-1}).

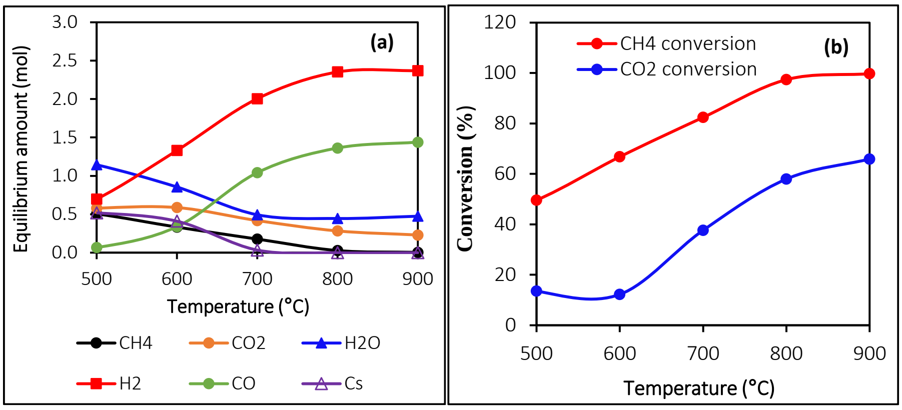

Figure S 5. Thermodynamic equilibrium at 1.5 bar total pressure of a mixture initially containing 1, 0.67, 0.09, and 0.85 mol per minute of CH_4, CO_2, O_2, and H_2O, respectively; (a) equilibrium amount of the main species considered; (b): methane and carbon dioxide conversion at equilibrium.

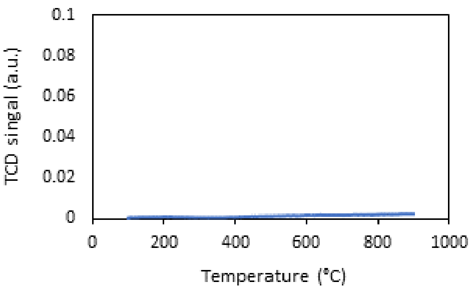

Figure S 6. TPO analysis under 5vol.%O_2/N_2 (5°C/min heating rate) of the used Ni/HAP_R800 catalyst, recovered after TRM test at 800 °C for 200 hours-on-stream.
